# Dual-Compartment Anti-Inflammatory and Chondroprotective Effects of Intra-Articular Hydrolyzed Collagen in Experimental Osteoarthritis

**DOI:** 10.3390/medicina61081461

**Published:** 2025-08-14

**Authors:** Mustafa Dinç, Ömer Cevdet Soydemir, Recep Karasu, Aysun Saricetin, Hunkar Cagdas Bayrak

**Affiliations:** 1 Orthopedics and Traumatology Clinics, Bursa City Hospital, 16250 Bursa, Turkey; dromer77@hotmail.com (Ö.C.S.); recepkarasu@hotmail.com (R.K.); 2 Department of Pathology, Faculty of Veterinary Medicine, Bursa Uludag University, 16059 Bursa, Turkey; aysunsaricetin@gmail.com; 3 Orthopedics and Traumatology Clinics, Çekirge Devlet Hastanesi, 16090 Bursa, Turkey; cagdasbayrak90@gmail.com

**Keywords:** osteoarthritis, intra-articular collagen, cartilage degradation, synovial inflammation, oxidative stress

## Abstract

*Background and Objectives*: Osteoarthritis (OA) is a degenerative joint disease involving inflammation, oxidative stress, and extracellular matrix (ECM) degradation, leading to cartilage damage and joint dysfunction. This study aimed to evaluate the chondroprotective effects of intra-articular hydrolyzed collagen in a rat model of knee OA using a dual-compartment biochemical and histological approach. *Materials and Methods*: Twenty male Sprague-Dawley rats underwent ACL transection to induce osteoarthritis and were randomly assigned to receive intra-articular hydrolyzed collagen or saline once weekly for three weeks. At six weeks, knee joints were evaluated histologically using the Mankin score. Synovial fluid and cartilage homogenates were analyzed via enzyme-linked immunosorbent assay (ELISA) for cytokines, cartilage degradation markers, and oxidative stress indicators. *Results*: The collagen-treated group demonstrated significantly lower Mankin scores. Levels of pro-inflammatory cytokines, interleukin-1 beta (IL-1β), interleukin-6 (IL-6), and tumor necrosis factor-alpha (TNF-α), as well as cartilage degradation markers, matrix metalloproteinase-13 (MMP-13), C-terminal crosslinked telopeptide of type II collagen (CTX-II), and cartilage oligomeric matrix protein (COMP), were significantly reduced (*p* < 0.05). Additionally, oxidative stress indicators including inducible nitric oxide synthase (iNOS), total oxidant status (TOS), and oxidative stress index (OSI) were decreased, while total antioxidant status (TAS) was increased in both synovial fluid and cartilage homogenates (*p* < 0.05). *Conclusions*: Intra-articular hydrolyzed collagen reduced inflammation, oxidative stress, and extracellular matrix (ECM) degradation, indicating potential chondroprotective effects across both synovial and cartilage compartments.

## 1. Introduction

Osteoarthritis (OA) is a progressive joint disease characterized by cartilage deterioration, synovial inflammation, and oxidative stress, resulting in pain and functional loss [1]. Current treatments like NSAIDs, corticosteroids, and hyaluronic acid primarily provide symptomatic relief but do not address the underlying pathology [2,3]. As a result, regenerative therapies, such as intra-articular collagen injections, are gaining attention for their potential chondroprotective, anti-inflammatory, and antioxidative effects [4,5].

Collagen is a key structural component in the extracellular matrix (ECM) of articular cartilage, with type II collagen maintaining cartilage strength and resilience. ECM degradation is mediated by pro-inflammatory cytokines such as interleukin-1 beta (IL-1β), interleukin-6 (IL-6), and tumor necrosis factor-alpha (TNF-α), which upregulate matrix metalloproteinases (MMPs), particularly MMP-13, leading to cartilage breakdown [6,7]. Oxidative stress further promotes chondrocyte senescence and apoptosis, exacerbating joint degeneration [8].

A growing body of clinical and preclinical studies has examined intra-articular collagen injections for OA. However, a critical gap remains in addressing the interplay between synovial inflammation and cartilage degradation. Synovial fibroblasts and macrophages secrete IL-1β, IL-6, and TNF-α, stimulating chondrocytes to produce MMP-13, leading to type II collagen and ECM breakdown [9]. Simultaneously, cartilage degradation products like c-terminal crosslinked Telopeptide of Type II Collagen (CTX-II) and cartilage oligomeric matrix protein (COMP) amplify synovial inflammation, creating a vicious cycle of joint destruction. Despite recognizing this synovial–cartilage crosstalk as central to OA progression, most therapies target either inflammation or degradation separately [10].

Hydrolyzed collagen consists of low-molecular-weight peptides (3–6 kDa) derived from enzymatic breakdown of native collagen, enhancing solubility, tissue diffusion, and biological activity compared to non-hydrolyzed forms [11,12,13]. Bovine-derived hydrolyzed collagen formulations have been developed for intra-articular use, delivering collagen directly into the joint space [14]. This localized administration supports cartilage homeostasis by supplying key amino acids (glycine, proline, hydroxyproline), stimulating chondrocytes to produce type II collagen and proteoglycans, and downregulating inflammatory mediators and matrix-degrading enzymes like MMP-13 [11,13,15,16].

Although intra-articular collagen injections have shown clinical promise for symptomatic relief in OA patients [17,18,19,20], their molecular mechanisms remain insufficiently characterized. Preclinical models, such as anterior cruciate ligament transection (ACLT)-induced OA, have provided insights into collagen’s chondroprotective potential, yet most studies emphasized systemic biomarkers over local joint effects. Direct analyses of synovial fluid and cartilage homogenates are limited, restricting understanding of localized actions. While some studies have reported reductions in inflammatory cytokines, oxidative stress markers, and ECM degradation markers, the results have been inconsistent, highlighting the need for further investigation [21,22,23,24].

In this study, we evaluated intra-articular hydrolyzed bovine collagen in a rat ACLT-induced OA model, hypothesizing that it would simultaneously reduce synovial inflammation and preserve cartilage integrity. We assessed histological and biochemical outcomes, including suppression of pro-inflammatory cytokines, oxidative stress markers, and cartilage degradation biomarkers in both synovial fluid and cartilage homogenates. This dual-compartment analysis offers novel insights into synovial–cartilage crosstalk and the potential chondroprotective role of intra-articular collagen in OA.

## 2. Materials and Methods

### 2.1. Animals

This study included twenty male Sprague-Dawley rats, aged 12 weeks and weighing 300–350 g at the time of procurement, obtained from the Experimental Animals Breeding and Research Unit of the Medical Faculty of Uludağ University. All animals were housed under controlled laboratory conditions with a 12 h light/dark cycle, regulated temperature (21 ± 1 °C) and humidity (55 ± 5%), and ad libitum access to food and water. Prior to experimental procedures, rats underwent a 7-day acclimatization period. Animals were randomly allocated into two groups (n = 10 each). All procedures were approved by the Institutional Animal Welfare Committee (Approval No: 2025-04/07, Date: 4 March 2025).

### 2.2. Rat Model of ACLT-Induced Osteoarthritis

Osteoarthritis (OA) was induced using an ACLT model, a validated method that disrupts joint biomechanics and accelerates cartilage degeneration [25,26,27]. Rats were anesthetized with 4% isoflurane for induction and 2% for maintenance. After medial parapatellar incision on the right knee joint, the anterior cruciate ligament was carefully transected with micro-scissors to destabilize the joint. The anterior drawer test was used to confirm complete ACLT [28]. The joint capsule and skin were sutured with absorbable sutures. A single intramuscular dose of cefazolin (25 mg/kg) was administered postoperatively. Rats were housed under standard conditions, monitored daily, and allowed unrestricted movement. Starting postoperative day 1, the control group received weekly intra-articular injections of 50 µL saline, while the collagen group received 50 µL saline containing 5 mg of hydrolyzed bovine collagen (Smartbone^®^, Mezzovico-Vira, Switzerland) for three weeks. The intra-articular dose of 5 mg hydrolyzed collagen in 50 µL saline was selected based on prior studies demonstrating therapeutic effects without joint toxicity [14,21]. The 50 µL volume was adopted from Aytekin et al. [29], ensuring physiologic compatibility with the rat knee joint without overflow or synovial disruption. All rats were euthanized under deep anesthesia at six weeks post-injection for tissue collection.

### 2.3. Tissue Processing and Histological Analysis

Tissue samples from the medial femoral condyles and medial tibial plateaus were harvested, fixed in 10% phosphate-buffered (PBS) formalin for one week, and decalcified in 3% formic acid for 5–7 days. Longitudinal sections were placed into tissue cassettes. Samples were sequentially dehydrated through graded ethanol (70%, 80%, and 90%) for one hour each, followed by two rounds of absolute ethanol for one hour each. Clearing was performed in two stages: first immersion in xylene I for one hour, followed by xylene II overnight (~12 h). Samples were then infiltrated with paraffin at 56 °C for two hours per cycle before embedding in metal molds.

Serial 5 µm sections were obtained using a rotary microtome (Leica RM2155, Leica Biosystems, Buffalo Grove, IL, USA). Hematoxylin and Eosin (H&E) staining assessed general histopathology, while Safranin-O staining evaluated proteoglycan content, following standard protocols. Slides were examined under a light microscope (Olympus CX41, Tokyo, Japan).

Cartilage was evaluated using the Modified Mankin scoring system [30], assessing structure (0–6 points), chondrocyte cellularity (0–3 points), proteoglycan content by Safranin-O staining (0–4 points), and tidemark integrity (0–1 point), with a total score range of 0 (normal) to 14 (severely degenerated). This system is widely validated for detecting early and advanced OA changes, with high interobserver reliability [30,31]. Two independent blinded observers performed scoring to ensure reproducibility.

### 2.4. Biochemical Analysis

#### 2.4.1. Synovial Fluid Collection and Processing

Synovial fluid was collected immediately after euthanasia using a validated joint lavage technique adapted from Barton et al. [32]. Due to the limited native fluid volume (5–10 µL), 50 µL of sterile phosphate-buffered saline (PBS) was perfused into the joint cavity and aspirated simultaneously using 27G needles (BD Microlance™, Becton Dickinson, Drogheda, Ireland). This yielded 60–80 µL of diluted synovial fluid per knee, consistently obtained across all rats. The collected samples, containing native synovial contents, allowed duplicate ELISA analyses of inflammatory cytokines, oxidative stress markers, and cartilage degradation biomarkers. After centrifugation at 3000× *g* for 10 min at 4 °C, supernatants were stored at −80 °C until analysis.

#### 2.4.2. Cartilage Homogenate Preparation

Articular cartilage was carefully dissected from the medial femoral condyles and medial tibial plateaus immediately after euthanasia, avoiding subchondral bone and soft tissue contamination. Approximately 2 mm thick cartilage slices were harvested under a stereomicroscope, flash-frozen in liquid nitrogen, and stored at −80 °C. Frozen samples were pulverized under liquid nitrogen using a precooled mortar and homogenized with cold PBS (pH 7.4, 1:10 *w*/*v*) using a digital ultrasonic homogenizer (Hielscher UP200St, Hielscher Ultrasonics GmbH, Teltow, Germany) at 3000 rpm for 3 min under chilled conditions. Homogenates were centrifuged at 12,000× *g* for 20 min at 4 °C (Eppendorf 5810R, Hamburg, Germany) [33]. The resulting supernatants were aliquoted and stored at −80 °C for biochemical analysis, following sterile, cold-chain protocols per the manufacturer’s instructions (Elabscience^®^, Signal Hill, CA, USA).

#### 2.4.3. ELISA Analysis of Synovial Fluid and Cartilage Homogenate

Cytokine and biochemical marker levels were quantified using rat-specific ELISA kits (Elabscience, Houston, TX, USA). Analyses were performed on synovial fluid and cartilage homogenate supernatants collected post-euthanasia and stored at −80 °C. Samples were thawed once and immediately analyzed to prevent protein degradation. Markers assessed included pro-inflammatory cytokines (IL-1β, IL-6, TNF-α), cartilage degradation markers (MMP-13, CTX-II, COMP), and the oxidative stress marker (iNOS). All assays were conducted in duplicate, following kit instructions without additional dilution. Absorbance at 450 nm was measured using a Thermo Scientific™ Multiskan™ FC reader (Waltham, MA, USA), and concentrations were calculated via SkanIt™ Software version 10.0. Each group included 10 rats (n = 10 per group), and all samples were analyzed in a single batch to reduce inter-assay variability. Standard curves achieved R^2^ values > 0.99, with intra-assay coefficients of variation < 10% [34].

#### 2.4.4. Assessment of Oxidative Stress in Synovial Fluid and Cartilage Homogenate

Oxidative stress in synovial fluid and cartilage homogenates was assessed by measuring total oxidant status (TOS) and total antioxidant status (TAS) and calculating the oxidative stress index (OSI) using fully automated colorimetric methods developed by Erel [35]. TOS was measured by the oxidation of ferrous to ferric ions forming a colored complex with xylenol orange, detected at 560 nm. TAS was assessed by the ability of antioxidants to decolorize the ABTS (2,2′-azino-bis (3-ethylbenzothiazoline-6-sulfonic acid)) radical cation, with absorbance measured at 660 nm. Both assays were conducted with Rel Assay Diagnostics kits (Mega Tip, Gaziantep, Turkey) on an automated analyzer. TOS was expressed as μmol H_2_O_2_ equivalents/L, TAS as mmol Trolox equivalents/L, and OSI was calculated as (TOS/TAS) × 100 as arbitrary units.

### 2.5. Statistical Analysis

All statistical analyses were conducted using IBM SPSS Statistics version 27.0 (IBM Corp., Armonk, NY, USA). The Shapiro–Wilk test was used to assess the normality of data distribution. Continuous variables with a normal distribution are presented as mean ± standard deviation (SD), while non-normally distributed variables are reported as median and interquartile range (IQR). Between-group comparisons were performed using the independent samples *t*-test for parametric data and the Mann–Whitney U test for non-parametric data.

To further evaluate intergroup differences, fold change values were calculated. For parametric variables, fold change was computed as the ratio of group means; for non-parametric variables, it was based on the ratio of group medians.

A priori power analysis was conducted using the resource equation method in accordance with ethical standards for animal studies. Post hoc power analysis was performed using Cohen’s d for *t*-tests and Z-values for Mann–Whitney U tests. All comparisons demonstrated large-to-very-large effect sizes, supporting the validity of the findings.

## 3. Results

**Figure 1:**
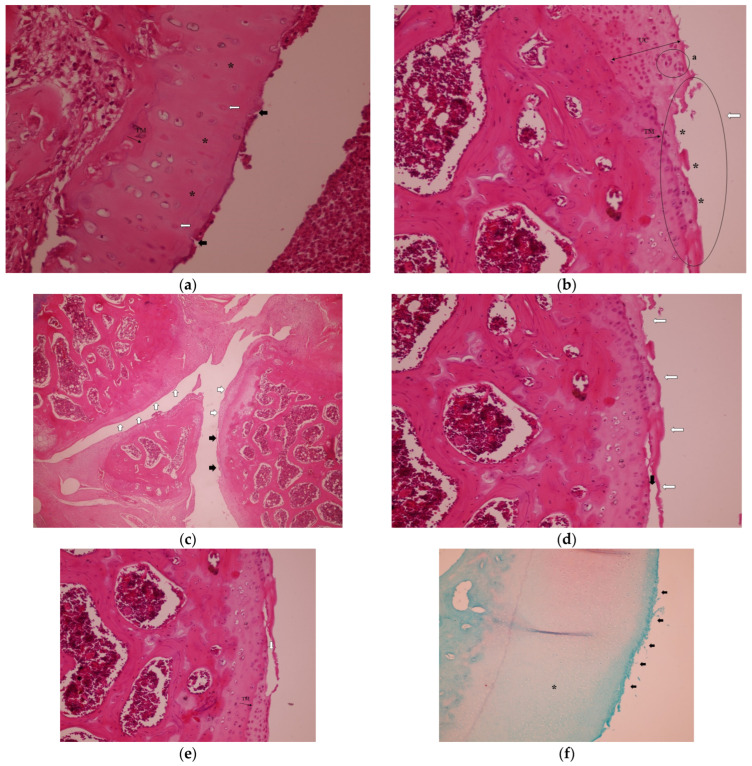
Representative histopathological images of the knee joint stained with hematoxylin–eosin (HE). In the Control group: (**a**) hypocellularity (*), some apoptotic chondrocytes (white arrows), cleft articular surface (black arrows) and tidemark irregularity, H&E, 40×, TM: Tidemark. (**b**) Loss of articular cartilage (*) and eroded articular surface (white arrow), chondrocyte clusters (a) H&E, 20×, TM: Tidemark. UC, uncalcified cartilage. (**c**) Irregular articular cartilage surface (white arrows) and loss of articular cartilage (black arrows), H&E, 4×. (**d**) Cleft (black arrow) and loss of articular cartilage, eroded articular surface (white arrows), H&E, 20×. (**e**) Cleft extending to the transitional zone (white arrow) and tidemark irregularity (black arrow), H&E, 20×, TM: Tidemark. (**f**) Safranin-O staining of articular cartilage. The asterisk-marked (*) areas indicate regions of decreased red staining, highlighting significant loss of proteoglycan content and eroded articular surface (black arrows) 10×.

In the Control group, histopathological analysis revealed tidemark irregularities and loss of articular cartilage integrity (Figure 1a), significant cartilage destruction, and chondrocyte clusters (a) (Figure 1b). Clefts extended into the transitional zone, with an irregular articular cartilage surface (white arrows) and loss of articular cartilage (black arrows) (Figure 1c). In the radial zone, clefts extended further (black arrow) (Figure 1d). Significant cartilage destruction was characterized by clefts extending into the transitional zone, irregularities in the tidemark, and disruption of the cartilage architecture (Figure 1e), as seen from Safranin-O staining of articular cartilage in the Control group. The asterisk-marked (*) areas indicate regions of decreased red staining, highlighting a significant loss of proteoglycan content (Figure 1f).

**Figure 2:**
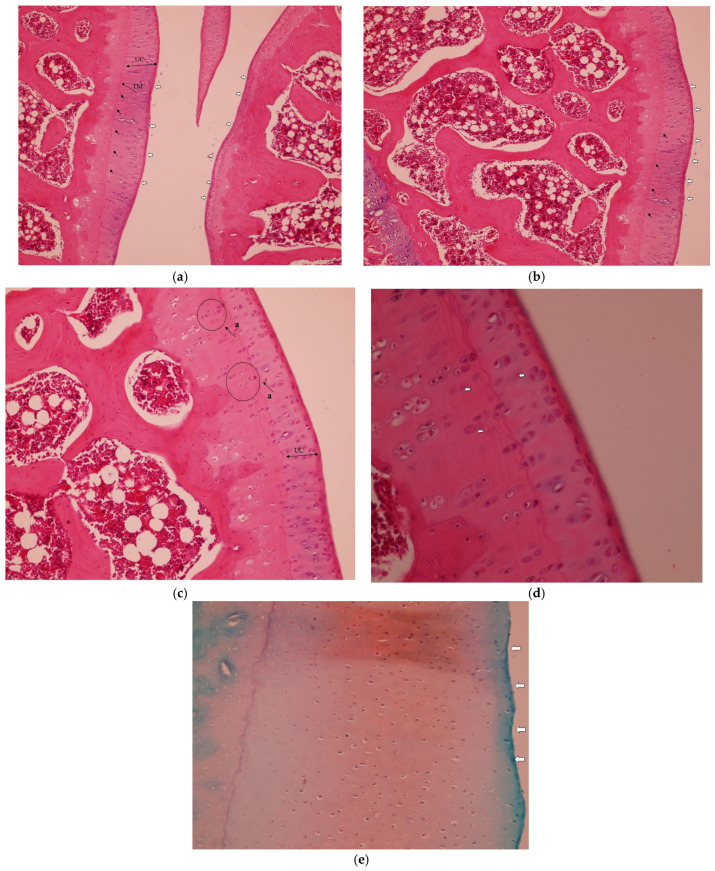
Representative histopathological images of the knee joint stained with hematoxylin–eosin (HE). In the Collagen group: (**a**) intact tidemark (black arrows) and intact articular cartilage (white arrows), H&E, 10×, TM: Tide Mark. UC, uncalcified cartilage. (**b**) Well-organized cellularity and intact tidemark (black arrows) and intact articular surface (white arrows), H&E, 20×. (**c**) No clefts and intact articular cartilage, H&E, 20×. UC, uncalcified cartilage. (a) Organized chondrocytes; (**d**) good cellularity, tidemark and articular surface with no clefts, H&E, 20×. The white arrow above the tidemark indicates organized chondrocytes in the deep cartilage layer, and the two arrows below indicate organized cells in the mineralized cartilage/subchondral area, some showing apoptotic features. (**e**) Safranin-O staining of articular cartilage. Intense and uniform red staining indicates preserved proteoglycan content within the cartilage matrix and regular articular surface 10×. White arrows indicate the smooth, intact articular surface without fibrillation or erosion.

In the Collagen group, the histopathological analysis demonstrated improved cartilage integrity with no clefts observed in any zone. The tidemark remained intact, and the articular surface showed no signs of disruption. Chondrocytes were well-organized and evenly distributed throughout the cartilage layers (Figure 2a–d), as seen from Safranin-O staining of articular cartilage. Intense and uniform red staining indicates preserved proteoglycan content within the cartilage matrix (Figure 2e).

In the histological evaluation, the collagen-treated group exhibited significantly lower scores across all Mankin scoring parameters—structural integrity, cellularity, Safranin-O staining, and tidemark integrity—compared to the control group (*p* < 0.001 for all), indicating marked histopathological improvement. Fold change analysis demonstrated a 2- to 3-fold reduction in most parameters in the collagen group, with the tidemark score reduced to zero in all treated samples, reflecting marked preservation of the cartilage–bone interface (Table 1).

### 3.1. Biochemical Analyses of Synovial Fluid

Biochemical analysis of synovial fluid revealed significantly decreased levels of pro-inflammatory cytokines (IL-1β, IL-6, TNF-α) and cartilage-degrading enzymes (MMP-13, CTX-II, COMP) in the collagen-treated group compared to controls (*p* < 0.001 for all). Additionally, oxidative stress markers including iNOS, TOS, and OSI were markedly reduced, while TAS was significantly elevated, indicating an improved oxidative–antioxidant balance. Notably, fold change values exceeded 2.0 for several critical biomarkers such as MMP-13, CTX-II, and iNOS, reinforcing the biochemical efficacy of intra-articular collagen administration (Table 2).

### 3.2. Biochemical Analyses of Cartilage Homogenate

Similarly, analysis of cartilage homogenates confirmed significant reductions in pro-inflammatory cytokines (IL-1β, IL-6, TNF-α) and cartilage degradation markers (MMP-13, CTX-II, COMP) in the collagen-treated group compared to controls (*p* < 0.001). Fold change analysis showed consistent reductions ranging from 1.6 to 2.0 across most markers. Additionally, oxidative stress parameters, including TOS and OSI, were significantly decreased, while TAS levels were elevated, further supporting the protective effect of intra-articular collagen on cartilage tissue integrity (Table 3).

## 4. Discussion

The findings of this experimental animal study demonstrate that intra-articular administration of hydrolyzed collagen exerts significant anti-inflammatory, antioxidative, and chondroprotective effects in a rat model of osteoarthritis. Histologically, the collagen-treated group exhibited significantly lower Mankin scores, with improved cartilage structure, reduced cellular disorganization, and preserved matrix staining. The absence of tidemark disruption in the collagen group further supports preservation of the osteochondral interface.

Biochemical analysis revealed consistent reductions in key pro-inflammatory cytokines (IL-1β, IL-6, TNF-α) and cartilage degradation biomarkers (MMP-13, CTX-II, COMP) in both synovial fluid and cartilage homogenates. MMP-13 plays a pivotal role in degrading type II collagen, the principal structural component of cartilage; thus, its suppression highlights hydrolyzed collagen’s role in preserving extracellular matrix (ECM) integrity and promoting chondroprotection [36,37]. Similarly, CTX-II is a degradation product of type II collagen, while COMP reflects overall cartilage turnover. The observed reductions in these biomarkers further indicate stabilization of the ECM and inhibition of catabolic activity within the joint [38,39].

Additionally, oxidative stress parameters were markedly improved in the collagen group. Decreased levels of iNOS, TOS, and OSI, along with increased TAS, indicate enhanced antioxidant capacity and restoration of redox balance within the joint environment. Given the established role of oxidative stress in promoting cartilage matrix breakdown and altering chondrocyte function, these findings further support the protective potential of hydrolyzed collagen in modulating the osteoarthritic joint environment.

Importantly, these improvements were consistently observed across both joint compartments—synovial fluid and cartilage tissue—highlighting that hydrolyzed collagen exerts a broad, compartment-spanning biological effect. This dual-compartment activity reinforces the therapeutic potential of hydrolyzed collagen to modulate the osteoarthritic microenvironment at multiple levels. The convergence of molecular and histological improvements observed in this study strongly suggests that intra-articular hydrolyzed collagen may serve as an effective chondroprotective agent, capable of attenuating key processes involved in cartilage degradation and joint degeneration.

Several clinical studies have investigated the role of intra-articular collagen injections in osteoarthritis treatment. Volpi et al. [17] and Borja-Flores et al. [18] reported significant improvements in pain, joint function, and mobility; however, these studies primarily focused on clinical outcomes without investigating underlying biochemical or structural changes. Consequently, the molecular mechanisms underlying these clinical benefits remain unclear. Similarly, Lee et al. [19] and Martin-Martin et al. [40] showed reductions in WOMAC and VAS scores after collagen injections yet did not assess inflammation, oxidative stress, or extracellular matrix (ECM) degradation, leaving the molecular basis of these benefits unexplored. Our study addresses this gap by providing direct biochemical and histological evidence that hydrolyzed collagen significantly reduces pro-inflammatory cytokines, oxidative stress markers, and cartilage degradation biomarkers, thereby linking molecular improvements to potential clinical outcomes. De Luca et al. [14] conducted a hybrid preclinical–clinical study showing increased type II collagen production after collagen use but similarly lacked analysis of inflammatory or oxidative stress markers. In contrast, our dual-compartment approach—analyzing both synovial fluid and cartilage homogenates—clearly demonstrates collagen’s modulation of inflammatory and catabolic pathways.

In addition, preclinical studies suggest intra-articular collagen supports cartilage repair, but most focus on systemic rather than localized joint effects. Naraoka et al. [22] reported no significant reduction in MMP-13 after collagen injection, whereas our study demonstrated significant decreases in MMP-13, CTX-II, and COMP, indicating superior ECM preservation. Similarly, Almonte-Becerril et al. [1] and Suh et al. [21] showed structural preservation of cartilage but did not assess oxidative stress or inflammatory markers. In contrast, our findings clearly demonstrate that hydrolyzed collagen reduces inflammatory mediators and oxidative stress, both critical factors in OA progression. De Luca et al. [14] and Furuzawa-Carballeda et al. [41] reported increased type II collagen synthesis and reduced IL-1β and TNF-α but lacked direct evaluation of cartilage degradation markers. By simultaneously assessing inflammation, oxidative stress, and ECM integrity, our study offers a more comprehensive and integrated understanding of collagen’s chondroprotective mechanisms in OA.

The protective effects of hydrolyzed collagen in OA likely arise from its combined anti-inflammatory, antioxidative, and ECM-stabilizing properties. Chronic inflammation and oxidative stress play central roles in OA progression by inducing synovial inflammation, ECM degradation, chondrocyte apoptosis, and cellular senescence [42,43]. While previous studies have documented the anti-inflammatory effects of hydrolyzed collagen, our study further demonstrates compartment-specific reductions in IL-1β, IL-6, and TNF-α within both synovial fluid and cartilage homogenates. This dual-compartment reduction is particularly significant, as chondrocytes actively contribute to OA progression by producing catabolic enzymes and inflammatory cytokines that accelerate cartilage degradation [44,45]. By lowering levels of these cytokines directly within cartilage, hydrolyzed collagen may reduce catabolic signaling, thereby protecting cartilage integrity at the cellular and molecular level.

Furthermore, synovial inflammation and cartilage degradation create a pathological feedback loop—synovial–cartilage crosstalk—whereby inflammatory cytokines upregulate MMP-13, while cartilage breakdown products amplify inflammation [46,47,48]. Our results suggest that hydrolyzed collagen disrupts this cycle by reducing both inflammatory mediators and ECM degradation markers. Oxidative stress also accelerates OA by promoting mitochondrial dysfunction and chondrocyte apoptosis [49]. The observed decrease in oxidative stress markers along with increased total antioxidant status suggests hydrolyzed collagen restores the oxidant–antioxidant balance crucial for maintaining cartilage health [8,50,51], as oxidative stress and chronic inflammation are strongly linked to chondrocyte senescence characterized by elevated IL-6, MMPs, and reactive oxygen species [52,53]. Hydrolyzed collagen may further safeguard cartilage by mitigating senescence-related deterioration. Thus, by simultaneously addressing synovial inflammation, oxidative stress, and cellular senescence, hydrolyzed collagen provides comprehensive protection to the joint environment, potentially preserving cartilage integrity and enhancing cell viability.

Our findings suggest that intra-articular hydrolyzed collagen represents a promising therapeutic approach for osteoarthritis (OA), particularly for addressing progressive joint degeneration. Unlike symptomatic treatments such as NSAIDs and corticosteroids, hydrolyzed collagen appears to confer structural and biochemical benefits by simultaneously targeting synovial inflammation, oxidative stress, and extracellular matrix (ECM) degradation. Given the persistent nature of these underlying pathological mechanisms in OA, an optimal therapeutic strategy should comprehensively address these interconnected processes. Our data demonstrate that intra-articular collagen effectively reduces pro-inflammatory cytokines, oxidative stress parameters, and cartilage degradation biomarkers, suggesting a multifaceted chondroprotective effect. These molecular-level improvements likely contribute to cartilage protection by mitigating inflammation-driven apoptosis and reducing cellular senescence, thereby supporting long-term joint health and functional integrity.

Although our experimental findings highlight the potential benefits of intra-articular collagen, translating these molecular improvements into meaningful clinical outcomes remains essential. Future clinical studies should investigate whether reductions in inflammation, oxidative stress, and cartilage breakdown biomarkers correspond to sustained improvements in pain, joint function, and cartilage preservation. Integrative approaches that combine biomarker analyses with patient-reported outcome measures, such as WOMAC and VAS scores, will be critical in establishing the clinical relevance of these molecular effects. Furthermore, comparative studies evaluating intra-articular collagen against other commonly used intra-articular therapies—including hyaluronic acid, corticosteroids, and platelet-rich plasma (PRP)—are warranted to clarify its therapeutic positioning. Such future clinical trials should ideally assess long-term efficacy, optimal dosing strategies, and whether molecular improvements correlate with symptomatic relief and improved functional outcomes in OA patients.

While our study demonstrates significant histological and biochemical improvements following intra-articular hydrolyzed collagen injections, several limitations must be acknowledged. First, although the ACLT-induced osteoarthritis model in rats is widely used and well-established, it does not fully replicate the complex biomechanical, cellular, and inflammatory environment of human OA. Therefore, validation in large-animal models and clinical trials across different disease stages is essential to enhance translational relevance. Second, this study focused on short-term outcomes following a single-dose administration. Long-term studies are needed to determine whether the observed reductions in inflammation, oxidative stress, and ECM degradation persist over time and whether these molecular effects translate into sustained cartilage preservation and functional improvement. Moreover, the optimal dosing strategy remains unclear. Future studies should investigate whether repeated intra-articular collagen injections are necessary, the ideal dosing intervals, and whether therapeutic effects plateau or regress after treatment cessation. Additionally, while improvements in cartilage structure were supported by lower Mankin scores and enhanced matrix staining, a direct analysis of collagen incorporation into native cartilage and ECM remodeling was not conducted. Further investigations employing labeled collagen peptides, histological tracking, and ultrastructural analysis are warranted to elucidate integration mechanisms. Lastly, our study did not explore intracellular signaling pathways or senescence-associated mechanisms that may underlie the observed effects. Future studies incorporating advanced molecular techniques—such as RT-PCR, Western blotting, and immunofluorescence—should aim to characterize the modulation of key regulatory pathways and the senescence-associated secretory phenotype (SASP), thereby providing deeper insights into the long-term chondroprotective potential of hydrolyzed collagen.

## 5. Conclusions

This study demonstrates that intra-articular hydrolyzed collagen provides significant short-term chondroprotective effects in a rat model of osteoarthritis by reducing inflammation, oxidative stress, and ECM degradation in both synovial fluid and cartilage. These dual-compartment improvements suggest a biologically active mechanism that supports cartilage preservation. Further studies are needed to evaluate long-term efficacy, dosing strategies, and underlying molecular pathways. With additional validation, hydrolyzed collagen may represent a promising biologically active agent for modifying OA progression.

## Figures and Tables

**Table 1:** Histopathological assessment of cartilage integrity in control and intraarticular collagen treatment groups.

Parameter	Control Group (Median [IQR])	Collagen Group (Median [IQR])	*p*-Value (Mann–Whitney U)	Fold Change (Control/Collogen
Structure	4 [1.25]	2 [1.00]	<0.001	2.0
Cellularity	3 [1.00]	1 [1.25]	<0.001	3.0
Safronin-O staining	2.50 [1.00]	2 [1.00]	0.005	1.25
Tidemark	1 [1.00]	0.00 [0.00]	0.001	∞
Total	10 [1.00]	5 [1.00]	<0.001	2.0

Data are presented as median [interquartile range]. Group comparisons were conducted using the Mann–Whitney U test. Fold change was calculated as the ratio of medians between the control and collagen groups.

**Table 2:** Biochemical comparison of synovial fluid parameters between control and intraarticular collagen-treated groups.

Synovial Fluid	Control Group N = 10	Collagen Group N = 10	*p*-Value	Fold Change (Control/Collogen)
Pro-inflammatory cytokine levels				
IL-1β (pg/mL)	1229.03 [73.95]	598.18 [74.25]	<0.001 *	2.05
IL-6 (pg/mL)	483.93 [32.54]	305.56 [51.45]	<0.001 *	1.58
TNF-α (pg/mL)	550.78 [62.82]	346.21 [47.24]	<0.001 *	1.59
Cartilage degrading markers				
MMP-13 (ng/mL)	14.77 [1.90]	6.38 [0.51]	<0.001 *	2.32
CTX-II (ng/mL)	6.51 [1.45]	2.54 [1.28]	<0.001 *	2.56
COMP (ng/mL)	76.47 [9.59]	41.08 [6.97]	<0.001 *	1.86
Oxidative stress markers				
iNOS (ng/mL)	43.05 [3.12]	14.21 [3.00]	<0.001 *	3.03
TAS (mMOL)	1.26 ± 0.02	1.42 ± 0.02	<0.001 **	0.89
TOS (µM H_2_O_2_ equiv.)	8.58 [1.01]	5.65 [0.41]	<0.001 *	1.52
OSI (oxidative stress index)	634.86 [94.99]	360.71 [54.80]	<0.001 *	1.76

Data are presented as mean ± standard deviation (SD) for TAS (total antioxidant status) and as median [interquartile range] for all other variables. * indicates Mann–Whitney U test used for non-parametric data. ** indicates independent samples *t*-test used for parametric data. Fold change values represent the ratio of control to collagen values (mean for parametric variables, median for non-parametric variables). Abbreviations: IL-1β, Interleukin-1beta; IL-6, Interleukin-6; TNF-α, Tumor Necrosis Factor alpha; MMP-13, Matrix Metalloproteinase-13; CTX-II, C-terminal cross-linking telopeptide of type II collagen; COMP, Cartilage Oligomeric Matrix Protein; iNOS, Inducible Nitric Oxide Synthase; TAS, total antioxidant status; TOS, total oxidant status; OSI, oxidative stress index.

**Table 3:** Biochemical analysis of cartilage homogenates comparing control and intra-articular collagen-treated groups.

Cartilage Homogenate	Control Group N = 10	Collagen Group N = 10	*p*-Value	Fold Change (Control/Collogen)
Pro-inflammatory cytokine levels				
IL-1β (pg/mL)	1084.29 [168.31]	655.90 [44.96]	<0.001 *	1.65
IL-6 (pg/mL)	508.21 [59.15]	285.33 [32.73]	<0.001 *	1.78
TNF-α (pg/mL)	603.03 [28.43]	349.72 [38.99]	<0.001 *	1.72
Cartilage degrading markers				
MMP-13 (ng/mL)	14.62 [2.40]	7.08 [1.58]	<0.001 *	2.06
CTX-II (ng/mL)	5.71 ± 0.38	2.81 ± 0.21	<0.001 **	2.03
COMP (ng/mL)	54.56 ± 1.65	34.24 ± 1.21	<0.001 **	1.59
Oxidative stress markers				
iNOS (ng/mL)	14.64 [1.67]	5.95 [0.48]	<0.001 *	2.46
TAS (mMOL)	1.02 [0.03]	1.32 [0.07]	<0.001 *	0.77
TOS (µM H_2_O_2_ equiv.)	6.70 ± 0.27	3.85 ± 0.20	<0.001 **	1.74
OSI (oxidative stress index)	629.10 [67.48]	310.53 [18.72]	<0.001 *	2.02

Data are presented as mean ± standard deviation (SD) for CTX-II, COMP, and TOS, and as median [interquartile range] for all other variables. * indicates Mann–Whitney U test used for non-parametric data. ** indicates independent samples *t*-test used for parametric data. Fold change values represent the ratio of control to collagen values (mean for parametric variables, median for non-parametric variables). Abbreviations: IL-1β, Interleukin-1beta; IL-6, Interleukin-6; TNF-α, Tumor Necrosis Factor alpha; MMP-13, Matrix Metalloproteinase-13; CTX-II, C-terminal cross-linking telopeptide of type II collagen; COMP, Cartilage Oligomeric Matrix Protein; iNOS, Inducible Nitric Oxide Synthase; TAS, total antioxidant status; TOS, total oxidant status; OSI, oxidative stress index.

## Data Availability

The data presented in this study are available on request from the corresponding author.
